# Vestigial Rides a Fat/Dachsous Wave in Wing Development

**DOI:** 10.1371/journal.pbio.1000389

**Published:** 2010-06-01

**Authors:** PLoS Biology Editors

**Affiliations:** PLoS Biology, Public Library of Science, San Francisco, California, United Stated of America

**Figure pbio-1000389-g001:**
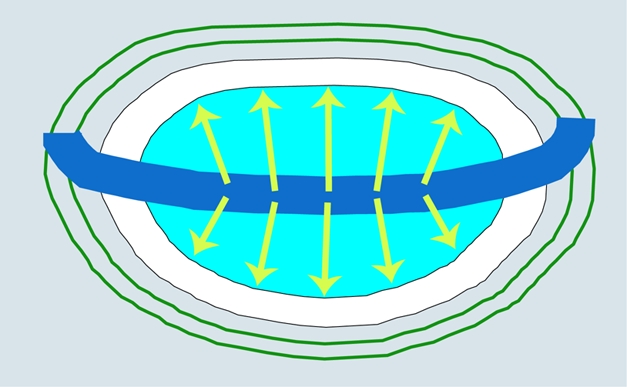
Border cells (blue) express Vestigial, secrete Wingless (green), and send a Vestigial-dependent “feed-forward” signal. Wingless and the feed-forward signal induce non-wing cells (white) to express Vestigial and become wing cells (turquoise). The process reiterates, promoting wing growth.

[Fig pbio-1000389-g001]One of the most enduring mysteries in biology concerns how tissue growth and pattern are controlled during development.

During fly development, many body segments contain disc-shaped pouches of cells known as imaginal discs, each containing the primordium of a specific adult limb (e.g., a wing or leg). The discs arise as small handfuls of embryonic cells, but soon thereafter undergo an explosion of growth and patterning governed by morphogens, signaling molecules that are secreted by special organizer cells within each disc. But how morphogens work, and especially how they control organ growth, remains a mystery.

The organizer cells that control wing growth are located at the boundary between the dorsal and ventral halves of the imaginal wing disc. Remarkably, these “border cells” exert their control through the agency of just two key genes. The first is *vestigial* (*vg*), which encodes a transcription factor and master regulator (Vestigial) that specifies the wing fate. The second is *wingless*, which encodes a morphogen (Wingless) that induces and sustains *vestigial* expression. In earlier work, Myriam Zecca and Gary Struhl showed that Wingless cannot act on its own to induce *vg*. Rather, it requires a second signal made by cells that already express *vestigial*. Thus, *vestigial*-expressing boundary cells send a “feed-forward” signal that acts together with Wingless to induce *vestigial* in neighboring cells, defining an inter-cellular circuit of *vestigial* auto-regulation that can propagate into surrounding tissue. In this issue of *PLoS Biology*, Zecca and Struhl identify the feed-forward signal and the molecular components of the circuit, providing new insight into this novel growth mechanism.

Expecting that the feed-forward signal should be active in wing cells, but not in the surrounding cells, Zecca and Struhl focused on specific cell adhesion molecules, the protocadherins names Fat and Dachsous. Fat is active in the wing primordium and Dachsous is active in the surrounding tissue. The molecules can act as ligands and receptors for each other. The authors find that Fat corresponds to the feed-forward signal sent by wing cells, and Dachsous to its receptor in non-wing cells. But surprisingly, they find that Fat is also required in non-wing cells to receive the feed-forward signal and that it is needed to transduce an opposing Dachsous signal. Thus, feed-forward signaling entails the generation of opposite Fat (wing) and Dachsous (non-wing) signals as well as the receptor activities of both proteins in receiving and transducing these signals.

Intriguingly, cells also need to sense opposing Fat and Dachsous signals to achieve proper polarization within the plane of the epithelium, a property called planar cell polarity. Hence Zecca and Struhl suggest that *vestigial* generates the feed-forward signal by regulating Fat and Dachsous so as to strongly polarize adjacent non-wing cells. In support, the authors also examine the atypical myosin Dachs and show that it is also essential for transduction of the feed-forward signal.

The authors have also filled in the rest of the circuit linking transduction of the feed-forward signal by Dachs to the transcriptional activation of *vestigial* and the generation of new feed-forward signal by the newly recruited wing cell. As in previous studies of growth, they find that Dachs acts by repressing a conserved tumor suppressor pathway involving the proteins Warts and Hippo, leading to a burst of activity of the transcriptional co-activator Yorkie. Yorkie in turn forms a complex with Scalloped, a DNA binding protein that directly activates *vestigial*.

Remarkably, Yorkie and Vestigial are known to function as interchangeable co-activators for Scalloped. Thus, once a cell starts making Vestigial, Vestigial can substitute for Yorkie to drive its own expression. This explains how *vestigial*-expressing border cells organize the long-range propagation of *vestigial* expression. First, they act on their immediate neighbors through the transient activation of Yorkie to induce *vestigial* expression, converting them into wing cells. Once converted, the new wing cells generate new feed-forward signal, repeating the cycle to recruit yet more wing cells. Crucially, cells must receive Wingless input for Scalloped to activate *vestigial* transcription, whether in conjunction with Yorkie or with Vestigial. Hence, as Wingless spreads outwards from border cells, it fuels a wave front of Fat–Dachsous signaling and Yorkie activation that leaves in its wake a growing population of Vestigial-expressing wing cells.

But the feed-forward mechanism explains only part of the story of how Wingless controls wing growth. Wingless is also required for Vestigial-expressing cells within the primordium to grow and proliferate. And it appears that wing cells, induced and maintained by Wingless signaling, generate an additional, unknown signal that stimulates the growth and proliferation of non-wing cells from which new wing cells will be recruited.

Many questions remain, not least of which is what ultimately limits the size of the wing and whether equivalent feed-forward mechanisms operate to promote the growth of other organs. Encouragingly, recent evidence suggests that the Warts–Hippo pathway and Yorkie may act through the regulation of cell fate to control organ size in vertebrate systems. Future work is needed, however, to identify the upstream signals and downstream master regulatory genes in these other systems.

**Zecca M, Struhl G (2010) A Feed-Forward Circuit Linking Wingless, Fat-Dachsous Signaling, and the Warts–Hippo Pathway to** ***Drosophila*** **Wing Growth. doi:10.1371/journal.pbio.1000386**

